# The relation between MIND diet with psychological disorders and psychological stress among Iranian adults

**DOI:** 10.1186/s12888-022-04128-2

**Published:** 2022-07-25

**Authors:** Roya Barkhordari, Mahdieh Namayandeh, Masoud Mirzaei, Mohammad Hassan Sohouli, Mahdieh Hosseinzadeh

**Affiliations:** 1 Department of Nutrition, School of Public Health, Shahid Sadoughi University of Medical Sciences, Yazd, Iran; 2 Center for Healthcare Data Modeling, Departments of Biostatistics and Epidemiology, School of Public Health, Shahid Sadoughi University of Medical Sciences, Yazd, Iran; 3 Yazd Cardiovascular Research Centre, Shahid Sadoughi University, of Medical Sciences, Yazd, Iran; 4 Student Research Committee, Department of Clinical Nutrition and Dietetics, Faculty of Nutrition and Food Technology, Shahid Beheshti University of Medical Sciences, Tehran, Iran; 5 Nutrition and Food Security Research Center, Shahid Sadoughi University of Medical Sciences, Yazd, Iran

**Keywords:** Mental Disorders, Depression, Anxiety, Mediterranean Diet, DASH

## Abstract

**Background:**

Given the role of dietary antioxidants in relieving depression, anxiety, and stress, as well as studies on the beneficial effects of the Mediterranean and Dash diets separately on these problems, in this study, we examine the relationship between the MIND index (Mediterranean- DASH diet Intervention for Neurodegenerative Delay) as a combined indicator of the Mediterranean and DASH diet with psychological disorders such as depression, anxiety, and psychological stress among a large sample of the Iranian adult population.

**Methods:**

This cross-sectional study was performed on 7165 participants of the enrollment phase of Yazd Health Study (YaHS) and Yazd Nutrition Study (TAMYZ) a valid 178-item semi-quantitative food frequency questionnaire (FFQ) was used to assess participants’ food intake. The MIND diet score was calculated based on participants’ dietary intakes obtained from FFQ. Also, the valid Iranian version of the Depression, Anxiety, and Stress Scale (DASS 21) was used to assess psychological disorders and stress. In addition, the association between the MIND diet and psychological disorders and stress was assessed through logistic regression.

**Results:**

The mean ± SD score was 3.33 ± 3.79 for depression, 2.99 ± 3.65 for anxiety, and 5.93 ± 4.70 for psychological stress. The mean score of MIND in this study was 7.56. After adjustment for after adjusting for age, gender, intake of energy, BMI, history of chronic disease, marital status, education level, smoking history, physical activity level, pregnancy or lactation, intakes of dietary EPA, DHA, and fiber, individuals in highest compared to the lowest quartile of MIND diet score had significantly lower odds of depression (OR = 0.62, 95% CI 0.40 – 0.96; P-trend = 0.02) and anxiety (OR = 0.61, 95% CI 0.41 – 0.91; P-trend = 0.01). However, no significant association was observed for psychological stress (OR = 0.57, 95% CI 0.28 – 1.14; P-trend = 0.83).

**Conclusion:**

Therefore, it seems that following the MIND diet can prevent the possibility of these psychological problems. However, there is a need to design studies with more robust methodologies such as clinical trial studies.

## Introduction

Psychological disorders and stress have caused great concern and burden among people, especially adult [1]. Depression and anxiety are among the most important psychological disorders and have been one of the causes of disability in the last 25 years [2]. Prevalence of mild, moderate, intense, and highly intense anxiety in Iran was 31, 37, 19, and 2, respectively [3]. In addition, the prevalence of depression was 44, the prevalence of stress was 40, the prevalence of overt anxiety was 21, and prevalence of covert anxiety was 24 [3]. However, conventional treatments have not been sufficient to prevent the spread of these patients [4].

In the last few decades, the effects of various factors, especially diet and the intake of certain nutrients on the nervous system and psychological disorders and stress have been considered [5]. So that, a higher intake of antioxidant micronutrients and some food groups such as fruits, vegetables, low-fat dairy products and lower intake of sugar-sweetened drinks and high-fat dairy products have been associated with a reduction in the risk of psychological [4, 5]. However, given that most of the nutrients and special food in the diet are usually consumed together and may have synergistic effects on each other, the examining of dietary patterns that reflect all the characteristics of the diet is more appropriate and accurate [6].

Evidence has shown that an unhealthy and western diet increases the risk of mental disorders and stress [7]. Also, in previous studies, the relationship between Mediterranean and DASH diets on psychological disorders and stress has been investigated, however, the results have been contradictory. In some studies, the beneficial effects of these diets on these patients were shown [8–10], but in other studies, there was insufficient evidence in this regard [11, 12]. In this context, a neuroprotective diet pattern has recently been described as the MIND (Mediterranean- DASH diet Intervention for Neurodegenerative Delay) diet, which is a combination of Mediterranean and DASH diets [4].

This diet pattern seems to play an important role in life stress and psychological health by emphasizing plant-based foods and limiting animal foods and saturated fats [13]. This diet pattern is also rich in antioxidant nutrients that reduce oxidative stress and inflammation as one of the important factors involved in neurodegenerative disease [14]. One study found that consumption MIND diet reduced the risk of Alzheimer’s disease [13]. Also in one study, adherence to the MIND diet further reduced cognitive impairment compared to the Mediterranean and DASH diets [15]. However, in one study, no association was found between MIND diet and depression [4].

Tus, regarding the important role of dietary intake in the prevention and management of psychiatric disorders as well as stress and little evidence linking MIND and these problems, the current cross-sectional study was performed to evaluate the relationship between MIND score with depression, anxiety and psychological stress among a large representative sample of Iranian adult population.

## Methods

This study was conducted in the framework of a prospective cohort study of Yazd Health Study (YaHS) with a focus on various determinants of health including nutrition. Yazd Health Study (YaHS) has been conducted since 2014 and examines the health status, chronic non-communicable diseases (NCDs), and related risk factors in adult residents of Yazd Greater Area aged 20–70 years. Also, in this study, the findings of the study of Taghzieh Mardom-e-Yazd (TAMYZ), which is a study in line with YaHS, have been used. Study design, sample selection, characteristics of participants in the study, as well as details of data collection methods, have been published in previous studies [16]. Data collection was done in two main stages among the adult population of Yazd province. In the first phase, a questionnaire including demographics, history of chronic diseases, anthropometric measurements, quality of life, psychological status, eating habits, surgical history, and women’s health was completed for 9965 people by trained interviewers (94.9% response rate), out of this number 4010 people gave blood test samples. In the second phase of the study, the nutritional health of the people entitled Taghzieh Mardom-e-Yazd (TAMYZ) was examined using a semi-quantitative food frequency questionnaire. Information on socio-demographic characteristics, tobacco use, history of chronic disease, psychological health, and physical activity assessments and dietary evaluation was obtained by a validated questionnaire. All participants signed an informed consent form before participating in the study. In this study, the subjects are selected based on the following inclusion and exclusion criteria:

Inclusion criteria include: 1. Age 70–20 years 2. Availability of nutritional information of participants in study 3. Availability of psychological health assessment data for participants 4. Individual consent to participate in the study. Exclusion criteria: 1. Have a history of diabetes, high blood pressure, heart disease, and cancer 2. Follow a special diet 3. Under and over-reporting (less than 800 kcal and more than 6500 kcal) 4. Pregnancy.

### Dietary assessment

The semi-quantitative FFQ was administered to assess the dietary foods and supplements. The original semi-quantitative FFQ contains 168 items, but 10 more questions were added on consumption of Yazd-specific frequently consumed food items, which made a total of 178 food items (included: breads and grains (*n* = 23); beans (*n* = 7); meats, fish, and shellfish (*n* = 19); milks and dairy products (*n* = 17); vegetables (*n* = 26); fruits (*n* = 40); fats and nuts (*n* = 13); beverages (*n* = 5); and snacks and sweets (*n* = 28). The semi-quantitative FFQ was previously validated for the Iranian population [16, 17], so the questionnaire was completed by trained interviewers. Participants were supposed to report the amount and frequency of consuming each food item per month, week, or day in the past year. Moreover, a food photograph book was used for all participants as a reference, so that they could estimate the portion size of foods as a unit accurately. Participants were also asked to report their intake frequency with regard to all food items based on 10 multiple-choice frequency response categories ranging from “never or less than once a month” to “10 or more times per day.” Later, the amount of food consumed at each intake was estimated using questions with five predefined answers. Frequency and usual amount of food items consumption were asked by participants and finally, amounts of intakes were converted to grams using guidelines of household scales [18].

### Calculate of the MIND diet score

In order to calculate the MIND diet score, FFQ questionnaire data were used. The MIND diet model was determined based on Morris’s study [13]. The components of MIND include 15 food items, 10 of which are known as healthy groups for the brain (green leafy vegetables, other vegetables, nuts, berries, beans, whole grains, fish, poultry, olive oil, and wine) and 5 of which are known as unhealthy food groups for the brain (red meats, butter and stick margarine, cheese, pastries and sweets, and fried/fast food). Each of these dietary components was obtained based on the total intake of the population for each item of FFQ. However, because 100% of the participants in the study reported being Muslim and not consuming wine, wine consumption was not included in the calculation of this dietary pattern. To calculate the score of this food pattern, first the participants in the study were classified based on tertile categories of food intake of 14 components of this diet pattern and then for healthy food components for the brain, the highest to lowest tertile of receiving these food items, scores 1, 2 and 3 were considered (respectively) and for unhealthy components, these scores were reversed and the lowest to highest tertile of receiving these food items, scores 1, 2 and 3 were considered, respectively. Finally, the higher the MIND score, the greater the adherence to this diet. The score range of this index is varied from 14 to 42. Then, by summarizing all these food items, the overall score of the MIND diet was obtained.

### Psychological health assessment (depression ‚anxiety and stress)

An Iranian version of the pre-completed DASS 21 questionnaire was used to assess psychological stress, depression, and anxiety [19]. The validity of DASS was measured using factor analysis and criterion validity (concurrent method). The correlation between the depression subscale and the beck depression inventory scale was + 0.70, between the anxiety subscale and Zung anxiety inventory was + 0.67, and between the stress subscale and perceived stress inventory was + 0.49. All correlations were significant. Males and females scores were significantly different on all subscales, therefore, separate norms for each gender were presented. This questionnaire consists of 3 subscales (stress ‚anxiety and depression) and 21 questions. Each of the subscales contains 7 questions, the final score of each of which was obtained through the sum of the scores of the related questions. Each question was scored from 0 (does not apply to me at all) to 3 (does not apply to me at all). Since DASS 21 is the short form of the DASS questionnaire (42 items), the accumulated scores for each subscale are multiplied by 2, and finally depression, anxiety, and stress were defined based on the scores obtained: depression: Score ≥ 10‚ anxiety: ≥ 8 points, and stress: ≥ 15 points.

### Anthropometric and physical activity assessment

The weight of the participants was measured using portable and digital scales (Omron BF511 inc. Nagoya, Japan) with an accuracy of 0.1 kg and while they only had one hand on light clothing, with the legs in the middle of the scales, slightly apart. standing height using a tape measure mounted on a flat wall without shoes with an accuracy of 0.1 cm while the heels, hips, and shoulders are attached to the wall, hands on both sides of the body and the head in the Frankfurt position were measured. Body mass index was calculated by dividing weight in kilograms by height squared in meters.

To evaluate physical activity, the Iranian version of the International Physical Activity Questionnaire (IPAQ) was used. Finally, physical activity is reported on a MET/ min/week basis [20].

### Statistical analyses

All statistical analyses were conducted using SPSS software (version 19.0; SPSS Inc, Chicago IL). The normality of variables was evaluated by Shapiro–Wilk tests. Comparisons across quartiles of MIND diet score was performed using one-way ANOVA. Mean values of more than two groups were assessed using Analysis of Variance (ANOVA) for normally distributed variables. Chi-square tests were used to compare categorical variables. The Binary Logistic regression models were used determine the separate association between MIND diet score and odds of psychological disorders and stress, in crude and covariate adjusted models. We adjusted the results in three models using a priori selected potential confounders. In the modified models, the confounders used a statistical and conceptual approach, respectively. In this way, variables with *P*value < 0.2 were considered as possible confounders and entered into logistic regression and the chances of disease were examined. Also, in the conceptual approach of modifying confounders in the second model, potential confounders were selected based on clinical concepts as well as based on previous articles and added to other confounders. The data were presented as mean ± standard deviation and, statistical significance was accepted, a priori, at *P* < 0.05.

The methodology of the current study was also ethically approved by the research ethics committee of Shahid Sadoughi University of Medical Sciences (approval code: IR.SSU.SPH.REC.1396.155).

## Result

### Study population characteristic

**Fig. 1:**
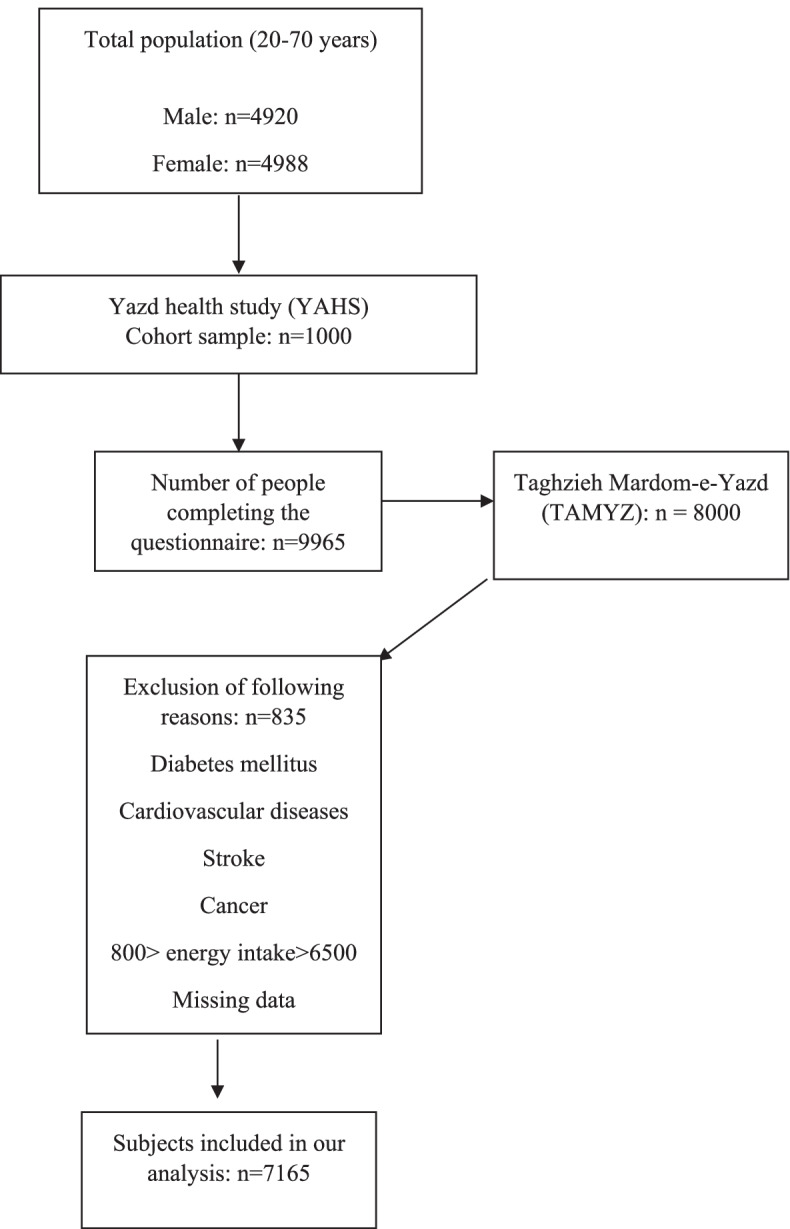
Flow chart of the participants and the final sample included

The mean ± SD for the BMI and physical activity of the study population was 27.00 ± 5.17 kg/m^2^ years and 901.16 ± 905.16 Met/min/week, respectively. The mean ± SD score were 3.33 ± 3.79 for depression, 2.99 ± 3.65 for anxiety, and 5.93 ± 4.70 for psychological stress. In this study, after considering the exclusion criteria, 7165 participants in the present study were included (Fig. 1). The mean score of MIND in this study was 7.56.

General and lifestyle characteristics of participants across quartiles of MIND diet score are presented in Table 1. Compared with those in the lowest quartiles of MIND diet score, subjects in the highest quartiles had higher BMI. In addition, a significant difference was observed for the variables of age, level of education, and Psychological health status of the participants among the quartiles of MIND diet score. No other significant difference was found in terms of general characteristics across quartiles of MIND diet score.

**Table 1:** General characteristics across quartiles of MIND diet score across phase 1 of Yazd Health Study (YaHS) participants 2014–2015

*Variables*	*Quartiles of MIND diet score*				
	***Q1***	***Q2***	***Q3***	***Q4***	***P***
***Age (years)***					
20–29	346(21.8)	514(32.4)	334(21.1)	392(24.7)	0.004
30–39	377(23.6)	527(33.0)	355(22.2)	339(21.2)	
40–49	306(19.3)	536(33.8)	384(24.2)	360(22.7)	
50–59	290(20.7)	441(31.5)	301(21.5)	370(26.4)	
60–69	233(19.2)	432(35.6)	281(23.1)	269(22.1)	
***Gender***					
Male	751(20.4)	1226(33.4)	819(22.3)	877(23.9)	0.584
Female	801(21.6)	1225(33.0)	834(22.5)	851(22.9)	
***BMI (kg/m2)***	26.94(5.12)	26.78(5.17)	27.09(5.11)	27.27(5.27)	0.019
***Physical activity (Met/min/week)***	906.60(859.83)	909.36(929.30)	893.85(919.74)	891.63(896.60)	0.910
***Education***					
***Lower than high school***	322(18.1)	571(32.2)	440(24.8)	443(24.9)	0.001
***High school***	485(23.2)	731(34.9)	436(20.8)	443(21.1)	
***Diploma and associated diploma***	509(22.2)	729(31.8)	510(22.3)	541(23.6)	
***Bachelors***	194(19.5)	327(32.9)	228(22.9)	246(24.7)	
***Masters and higher***	37(18.0)	74(36.1)	40(19.5)	54(26.3)	
***Smoking status***					
***Never smoker***	1342(21.2)	2082(32.9)	1389(22.0)	1515(23.9)	0.056
***Current smoker***	156(20.4)	267(34.9)	195(25.5)	148(19.3)	
***Ex‑smoker***	23(20.4)	40(35.4)	28(24.8)	22(19.5)	
***Marriage status***					
***Single***	169(19.7)	270(31.5)	186(21.7)	231(27.0)	0.294
***Married***	1330(21.2)	2091(33.4)	1407(22.5)	1436(22.9)	
***Widowed or divorced***	48(20.0)	79(32.9)	54(22.5)	59(24.6)	
***Psychological health status***					
***Depression***					
***Yes***	134(23.2)	218(37.7)	121(20.9)	105(18.2)	0.004
***No***	1365(20.7)	2165(32.8)	1479(22.4)	1582(24.0)	
***Anxiety***					
***Yes***	178(23.6)	272(36.1)	164(21.8)	140(18.6)	0.003
***No***	1321(20.6)	2111(32.9)	1436(22.4)	1547(24.1)	
***Stress***					
***Yes***	47(19.7)	99(41.6)	52(21.8)	40(16.8)	0.018
***No***	1452(20.9)	2284(33.0)	1548(22.3)	1647(23.8)	

Data are presented as mean (standard deviation (SD)) or number (percent)

*Abbreviations: BMI* Body mass index, *MET* Metabolic equivalent, *MIND diet* Mediterranean-DASH Intervention for Neurodegenerative Delay

^a^ Obtained from ANOVA or Chi-square test, where appropriate

### MIND diet and micro and macro nutrient intakes

Micro and macro nutrient intakes of study participants across quartiles of MIND diet score are showen in Table 2. Individuals in the highest quartiles of MIND diet score consumed higher vegetables, fruits, dairy (low and high fat dairy), legumes, nuts, whole grains, poultry, eggs, fish, energy, protein, carbohydrate, EPA, DHA, calcium, zinc, Potassium, Vitamin B12, vitamin E, vitamin D, vitamin C, folate, and fiber as well as a lower intake of red meat, processed meat, refine grain, cholesterol, sodium, and iron compared with those in the bottom quartiles.

**Table 2:** Dietary and nutrient intakes of study participants across Quartiles of MIND diet score

***Variables***	***Quartiles of MIND diet score***				***P***
	***Q1***	***Q2***	***Q3***	***Q4***	
***Food groups (g/day)***					
Fruits	408.70(358.65)	477.14(486.94)	516.45(481.83)	563.17(564.68)	< 0.001
Vegetables	228.32(8.88)	279.34(11.40)	317.28(10.47)	434.55(16.29)	< 0.001
Red meat	63.54(78.08)	54.45(57.59)	52.27(54.84)	49.23(54.86)	< 0.001
Processed meat	17.36(49.90)	13.77(36.13)	12.30(32.97)	16.08(42.31)	0.001
Low fat dairy product	36.63(83.37)	53.90(110.74)	67.83(117.72)	79.30(144.69)	< 0.001
High fat dairy product	184.29(255.85)	184.86(174.27)	176.83(163.67)	198.41(196.66)	0.013
Legumes	34.06(45.70)	45.02(60.79)	55.36(74.01)	58.07(62.14)	< 0.001
Nut	20.30(38.93)	24.23(38.31)	26.13(42.33)	26.28(38.40)	< 0.001
Whole grains	60.59(72.19)	69.00(73.07)	78.98(71.29)	92.73(73.01)	< 0.001
Refined grains	244.56(188.87)	225.79(181.76)	217.66(191.01)	200.93(157.71)	< 0.001
Poultry	30.76(49.55)	47.60(75.64)	61.71(83.95)	89.71(106.07)	< 0.001
Fish	11.58(33.93)	20.11(64.39)	22.05(46.36)	34.29(68.22)	< 0.001
Eggs	24.93(45.47)	31.00(66.36)	32.09(47.61)	31.00(43.78)	< 0.001
***Nutrients***					
Energy (Kcal/d)	2738.11(1390.48)	2863.49(1398.03)	2890.78(1353.42)	3094.41(1341.56)	< 0.001
Protein (g/d)	95.94(49.42)	108.37(57.63)	116.99(58.27)	135.81(67.24)	< 0.001
Fat (g/d)	110.63(75.64)	112.90(78.39)	110.67(70.32)	113.00(63.40)	0.607
Carbohydrate (mg/d)	388.59(224.94)	402.78(223.15)	404.42(211.14)	431.80(202.31)	< 0.001
Cholesterol	458.92(407.12)	395.88(379.20)	381.77(421.75)	343.68(320.69)	< 0.001
EPA (g/d)	0.01(0.04)	0.02(0.08)	0.03(0.08)	0.03(0.08)	< 0.001
DHA (g/d)	0.04(0.12)	0.06(0.22)	0.08(0.21)	0.09(0.22)	< 0.001
Calcium (mg/d)	869.00(489.23)	945.75(478.25)	985.32(47.78)	1072.49(497.73)	< 0.001
Iron (mg/d)	50.55(56.03)	48.42(92.25)	42.05(105.84)	31.30(41.67)	< 0.001
Sodium (mg/d)	5701.07(4610.26)	5598.69(4066.36)	5321.54(5931.77)	4664.84(5198.98)	< 0.001
Zinc(mg/d)	10.80(5.54)	11.52(5.81)	12.10(5.96)	13.43(6.53)	< 0.001
potassium(mg/d*)*	3531.50(1747.81)	3997.62(2212.91)	4235.13(2280.87)	4815.62(2525.60)	< 0.001
Vitamin B12 (mcg/d)	5.40(5.67)	5.80(6.71)	6.00(5.69)	7.12(6.73)	< 0.001
Vitamin E (mg/d)	10.04(9.89)	11.50(12.07)	11.90(12.38)	11.82(11.74)	< 0.001
Folate (mcg/d)	302.09(170.21)	359.83(213.18)	401.03(234.17)	458.46(258.48)	< 0.001
Vitamin D (mcg/d)	1.32(2.62)	1.40(1.82)	1.48(1.79)	1.74(2.19)	< 0.001
Vitamin C (mg/d)	169.62(136.77)	198.83(182.54)	214.34(172.90)	260.79(235.57)	< 0.001
Dietary fibre (g/day)	24.99(24.11)	27.63(25.18)	27.66(20.36)	31.70(23.11)	< 0.001

Data are presented as mean ± standard error (SE)

*Abbreviations: PUFA* Poly-unsaturated fatty acid, *SSB* Sugar-sweetened beverage, *SFA* Saturated fatty acid, *MIND diet* Mediterranean-DASH Intervention for Neurodegenerative Delay

^a^ Obtained from ANOVA

### MIND diet with psychological disorders and psychological stress

Multivariable-adjusted odds ratios (ORs) and 95% CI for psychological disorders and stress across quartiles of MIND diet score are shown in Table 3. In the crude and adjusted model 1 there was a evidence of decreased odds of depression and anxiety for subjects the highest compared to the lowest quartile of the MIND diet score (OR = 0.67, 95% CI 0.44 – 1.03; P-trend = 0.054 and OR = 0.64, 95% CI 0.42 – 0.99; P-trend = 0.037, respectively for depression and OR = 0.66, 95% CI 0.45 – 0.97; P-trend = 0.034 and OR = 0.63, 95% CI 0.43 – 0.93; P-trend = 0.021, respectively for anxiety). Furthermore, after adjusting for potential confounders in the model 2 and the final model, there was evidence that the odds of depression anxiety decreased with increasing categories of the MIND diet score (OR = 0.63, 95% CI 0.41 – 0.97; P-trend = 0.030 and OR = 0.62, 95% CI 0.40 – 0.96; P-trend = 0.024, respectively for depression and OR = 0.62, 95% CI 0.42 – 0.92; P-trend = 0.017 and OR = 0.61, 95% CI 0.41 – 0.91; P-trend = 0.014, respectively for anxiety).

**Table 3:** Odds ratio (95% CI) and 95% Confidence Interval (CI) for psychological disorders and stress according to Quartiles of MIND diet score

	***Quartiles of MIND diet score***				
	***Q1***	***Q2***	***Q3***	***Q4***	***P-trend***
***Depression***					
***Crude***	1.00 (Ref)	1.09(0.77–1.56)	1.00(0.67–1.47)	0.67(0.44–1.03)	0.054
***Model 1*** ^a^	1.00 (Ref)	1.06(0.74–1.51)	0.97(0.65–1.44)	0.64(0.42–0.99)	0.037
***Model 2*** ^b^	1.00 (Ref)	1.08(0.75–1.54)	0.98(0.66–1.45)	0.63(0.41–0.97)	0.030
***Model 3*** ^c^	1.00 (Ref)	1.08(0.75–1.54)	0.96(0.64–1.44)	0.62(0.40–0.96)	0.024
***Anxiety***					
***Crude***	1.00 (Ref)	1.01(0.73–1.39)	0.95(0.67–1.36)	0.66(0.45–0.97)	0.034
***Model 1*** ^a^	1.00 (Ref)	0.98(0.71–1.35)	0.93(0.65–1.33)	0.63(0.43–0.93)	0.021
***Model 2*** ^b^	1.00 (Ref)	0.99(0.72–1.37)	0.93(0.65–1.33)	0.62(0.42–0.92)	0.017
***Model 3*** ^c^	1.00 (Ref)	0.99(0.71–1.38)	0.92(0.64–1.32)	0.61(0.41–0.91)	0.014
***Stress***					
***Crude***	1.00 (Ref)	1.49(0.89–2.48)	1.08(0.60–1.93)	0.58(0.29–1.15)	0.056
***Model 1*** ^a^	1.00 (Ref)	1.44(0.86–2.41)	1.05(0.58–1.88)	0.56(0.28–1.11)	0.043
***Model 2*** ^b^	1.00 (Ref)	1.47(0.88–2.46)	1.05(0.58–1.89)	0.55(0.27–1.09)	0.035
***Model 3*** ^c^	1.00 (Ref)	1.50(0.89–2.53)	1.11(0.61–2.02)	0.57(0.28–1.14)	0.832

^a^Model 1: adjusted for age, gender, and intake of energy

^b^Model 2: adjusted for model 2 and BMI

^c^ Model 3: adjusted for model 2 and history of chronic disease (yes/no); marital status (single, married, widow or discovered); education level (lower than high school, high school, Diploma and associated diploma, Bachelors, Masters and higher); smoking history (never smoker, current smoker, ex-smoker); physical activity level (MET/min/week); pregnancy or lactation (yes/no); intakes of dietary EPA, DHA, and fiber (continues, g/d)

Although after adjusting for potential confounders, in models one and two, a significant relationship was observed between the reduced the odds of psychological stress and MIND diet score (OR = 0.56, 95% CI 0.28 – 1.11; P-trend = 0.043 and OR = 0.55, 95% CI 0.27 – 1.09; P-trend = 0.035, respectively), but in the final adjusted model, no significant relationship was observed between the odds of psychological stress and MIND diet score (OR = 0.57, 95% CI 0.28 – 1.14; P-trend = 0.832) after adjusting for age, gender, intake of energy, BMI, history of chronic disease, marital status, education level, smoking history, physical activity level, pregnancy or lactation, intakes of dietary EPA, DHA, and fiber.

## Discussion

In the present study, we investigated the relationship between adherence to MIND diet and the odds of developing psychological stress and psychological disorders among Iranian adults. The results of our study showed that after adjusting the possible confounders, the odds of depression and anxiety significantly decreased with increasing adherence to the MIND diet, however, no significant relationship was observed between dietary pattern and odds of psychological stress.

Recently, a cross-sectional study conducted by Salari Moghadam et al. [21] in 2019 on 3176 Iranian adults to investigate the relationship between this dietary pattern and the odds of psychological disorders and stress, showed that although the likelihood of depression and psychological stress decreases with increasing adherence to this diet, but contrary to our study, no significant effect was observed between anxiety and this dietary pattern that this difference in results could be due to differences in FFQ and psychological health assessment questionnaire used as well as differences in the score of these mental disorders.

In addition, contrary to our findings, a prospective study by Farsan et al. in 2018 reported no significant association between adherence to the MIND diet and the risk of depression. However, the components they used to build the MIND diet were slightly different from ours [4]. Previous studies, however, have shown an inverse relationship between foods rich in fruits and vegetables, legumes, and olives as components of the MIND diet with depression and anxiety [22, 23].

Some other studies have examined the DASH and Mediterranean diets as two components of the MIND diet. For example, in a study aimed at investigating the relationship between adherence to the Mediterranean diet and the prevalence of psychological disorders and stress among a large population of Iranian adults, the findings of this study showed that after controlling potential confounders, participants who followed the Mediterranean diet the most were less likely to suffer from depression, anxiety, and stress [24]. They also reported that high consumption of fruits and vegetables was associated with a lower risk of depression, anxiety, and psychological stress. In contrast, high cereal intake was positively associated with depression, anxiety, and psychological stress.

A cross-sectional analysis of 1183 data from 40- to 65-year-old Australian adults examined the association between cognitive impairment, memory, anxiety, stress, self-esteem, general health, and physical function following the Mediterranean diet [25]. The majority of Australians (71.7%) had a moderate adherence to the Mediterranean diet pattern and generally did not associate adherence to the Mediterranean diet with cognitive function. However, diet-related plant foods were negatively correlated with physical function and general health, and with perceived trait anxiety, depression, and stress. The important point in this study is that a significant portion of the diet in this Australian sample is of foods that are not normally part of the Mediterranean diet.

In another cross-sectional study of 181 girls between the ages of 18 and 25, the results showed that those with the highest levels of DASH adherence (highest adherence) had significantly lower scores on psychological stress and increased cognitive function [26].

Another article in line with our findings, aimed at examining the relationship between DASH diet and psychological health among 3846 Iranian adults, reported that moderate adherence to the DASH diet was associated with a lower likelihood of depression compared to those with the lowest adherence [12]. In addition, after controlling for potential confounders, an inverse relationship was observed between high DASH diet adherence and anxiety in participants. However, no significant relationship was reported between DASH diet intake and psychological stress.

In general, differences in findings between our study and other studies may be related to differences in sample size, characteristics, and health status of participants, as well as tools for assessing food intake and mental health, as well as calculating MIND diet scores.

The mechanisms by which the MIND diet may affect brain health are not yet fully understood by studies and scientists. However, given that inflammation and oxidative stress are important contributors to neurodegenerative diseases and mental disorders, it seems that the MIND diet may be through rich sources of vitamins, minerals, flavonoids and antioxidants [14, 27]. It has antioxidant and anti-inflammatory properties and thus protects the brain [27]. Berries and green leafy vegetables are two special components of this diet that berries have been proven to improve motor function (coordination and balance) in mice and increase speed and reduce walking error in humans [14]. This diet is also rich in carotenoids and vitamin E has been shown to be associated with a reduced risk of developing Parkinson’s and improved physical function in adults [14]. In addition, the MIND diet contains limited amounts of unhealthy foods including red meat and products, butter, fast foods and sweets that may be harmful to brain health by increasing inflammation and oxidative stress [13].

Among the strengths of the present study include: having a sufficient and large sample size, as well as controlling potential disruptive factors, using a validated 178-item semi-quantitative FFQ by a face-to-face interview and also using nutritionists to complete the questionnaires. However, due to the cross-sectional design of this study, the inference of causal relationships between this dietary pattern and mental disorders is affected. For example, poor diet may expose people to depression and mental stress. On the other hand, people who are depressed may consume large amounts of sweets and sugary foods to reduce depressive symptoms. Therefore, prospective studies are needed to confirm our findings. While to confirm these results, observational studies must be repeated in several parts of the world. However, it should be borne in mind that proper cross-sectional data analysis is a valuable first step in identifying the relationship between diet and disease. In addition, prospective cohort studies and clinical trials have their own weaknesses. Questionnaire-based data were also used to assess psychological disorders in this study. Although we used a valid Iranian version of the questionnaires, the possibility of incorrectly classifying participants in terms of results is also inevitable. In addition, we had no information about wine in our FFQ. Therefore, we removed this component in the construction of the MIND diet.

## Conclusion

Finally, our study showed that increasing adherence to the MIND diet may reduce the odds of depression and anxiety by increasing healthy foods with high levels of antioxidants. Therefore, by recommending this diet, you can prevent the possibility of these psychological problems. However, there is a need to design studies with stronger methodologies such as clinical trial studies.

## Data Availability

The data set generated and / or analyzed during the current study is not available to the public due to the non-publicity of the data, but is available at the reasonable request from corresponding author.

## References

[1] Auerbach RP, Mortier P, Bruffaerts R, Alonso J, Benjet C, Cuijpers P (2018). WHO World Mental Health Surveys International College Student Project: prevalence and distribution of mental disorders. J Abnorm Psychol.

[2] Teachman BA, McKay D, Barch DM, Prinstein MJ, Hollon SD, Chambless DL (2019). How psychosocial research can help the National Institute of Mental Health achieve its grand challenge to reduce the burden of mental illnesses and psychological disorders. Am Psychol.

[3] Valizadeh R, Sarokhani D, Sarokhani M, Sayehmiri K, Ostovar R, Angh P (2016). A study of prevalence of anxiety in Iran: systematic review and meta-analysis. Der Pharma Chemica.

[4] Fresán U, Bes-Rastrollo M, Segovia-Siapco G, Sanchez-Villegas A, Lahortiga F, de la Rosa P-A (2019). Does the MIND diet decrease depression risk? A comparison with Mediterranean diet in the SUN cohort. Eur J Nutr.

[5] Hosseinzadeh M, Vafa M, Esmaillzadeh A, Feizi A, Majdzadeh R, Afshar H (2016). Empirically derived dietary patterns in relation to psychological disorders. Public Health Nutr.

[6] Kim MK, Sasaki S, Otani T, Tsugane S, Group JPHCbPS (2005). Dietary patterns and subsequent colorectal cancer risk by subsite: a prospective cohort study. Int J Cancer.

[7] Khalid S, Williams CM, Reynolds SA (2016). Is there an association between diet and depression in children and adolescents? A systematic review. Br J Nutr.

[8] Rienks J, Dobson A, Mishra G (2013). Mediterranean dietary pattern and prevalence and incidence of depressive symptoms in mid-aged women: results from a large community-based prospective study. Eur J Clin Nutr.

[9] Sánchez-Villegas A, Henríquez-Sánchez P, Ruiz-Canela M, Lahortiga F, Molero P, Toledo E (2015). A longitudinal analysis of diet quality scores and the risk of incident depression in the SUN Project. BMC Med.

[10] Omar SH (2019). Mediterranean and MIND diets containing olive biophenols reduces the prevalence of Alzheimer’s disease. Int J Mol Sci.

[11] Quirk SE, Williams LJ, O’Neil A, Pasco JA, Jacka FN, Housden S (2013). The association between diet quality, dietary patterns and depression in adults: a systematic review. BMC Psychiatry.

[12] Valipour G, Esmaillzadeh A, Azadbakht L, Afshar H, Hassanzadeh A, Adibi P (2017). Adherence to the DASH diet in relation to psychological profile of Iranian adults. Eur J Nutr.

[13] Morris MC, Tangney CC, Wang Y, Sacks FM, Bennett DA, Aggarwal NT (2015). MIND diet associated with reduced incidence of Alzheimer’s disease. Alzheimers Dement.

[14] Agarwal P, Wang Y, Buchman A, Holland T, Bennett D, Morris M (2018). MIND diet associated with reduced incidence and delayed progression of Parkinsonism in old age. J Nutr Health Aging.

[15] Morris MC, Tangney CC, Wang Y, Sacks FM, Barnes LL, Bennett DA (2015). MIND diet slows cognitive decline with aging. Alzheimers Dement.

[16] Mirzaei M, Salehi-Abargouei A, Mirzaei M, Mohsenpour MA (2018). Cohort Profile: The Yazd Health Study (YaHS): a population-based study of adults aged 20–70 years (study design and baseline population data). Int J Epidemiol.

[17] Esfahani FH, Asghari G, Mirmiran P, Azizi F (2010). Reproducibility and relative validity of food group intake in a food frequency questionnaire developed for the Tehran Lipid and Glucose Study. J Epidemiol.

[18] Ghaffarpour M, Houshiar-Rad A, Kianfar H. The manual for household measures, cooking yields factors and edible portion of foods. Tehran: Nashre Olume Keshavarzy. 1999;7(213):42–58.

[19] Sahebi A, Asghari MJ, Salari RS (2005). Validation of depression anxiety and stress scale (DASS-21) for an Iranian population.

[20] Moghaddam MB, Aghdam FB, Jafarabadi MA, Allahverdipour H, Nikookheslat SD, Safarpour S (2012). The Iranian Version of International Physical Activity Questionnaire (IPAQ) in Iran: content and construct validity, factor structure, internal consistency and stability. World Appl Sci J.

[21] Salari-Moghaddam A, Keshteli AH, Mousavi SM, Afshar H, Esmaillzadeh A, Adibi P (2019). Adherence to the MIND diet and prevalence of psychological disorders in adults. J Affect Disord.

[22] Brookie KL, Best GI, Conner TS (2018). Intake of raw fruits and vegetables is associated with better mental health than intake of processed fruits and vegetables. Front Psychol.

[23] Liu M-w, Chen Q-t, Towne SD, Zhang J, Yu H-j, Tang R (2020). Fruit and vegetable intake in relation to depressive and anxiety symptoms among adolescents in 25 low-and middle-income countries. J Affect Disord.

[24] Sadeghi O, Keshteli AH, Afshar H, Esmaillzadeh A, Adibi P (2021). Adherence to Mediterranean dietary pattern is inversely associated with depression, anxiety and psychological distress. Nutr Neurosci.

[25] Crichton GE, Bryan J, Hodgson JM, Murphy KJ (2013). Mediterranean diet adherence and self-reported psychological functioning in an Australian sample. Appetite.

[26] Saharkhiz M, Khorasanchi Z, Karbasi S, Jafari-Nozad AM, Naseri M, Mohammadifard M (2021). The association between adherence to a dietary approaches to stop hypertension (DASH) diet and neuro-psychological function in young women. BMC nutrition.

[27] Salim S (2014). Oxidative stress and psychological disorders. Curr Neuropharmacol.

